# Analyzing Brain Functions by Subject Classification of Functional Near-Infrared Spectroscopy Data Using Convolutional Neural Networks Analysis

**DOI:** 10.1155/2016/1841945

**Published:** 2016-10-31

**Authors:** Satoru Hiwa, Kenya Hanawa, Ryota Tamura, Keisuke Hachisuka, Tomoyuki Hiroyasu

**Affiliations:** ^1^Faculty of Life and Medical Sciences, Doshisha University, Kyoto, Japan; ^2^Graduate School of Life and Medical Sciences, Doshisha University, Kyoto, Japan; ^3^DENSO CORPORATION, Aichi, Japan

## Abstract

Functional near-infrared spectroscopy (fNIRS) is suitable for noninvasive mapping of relative changes in regional cortical activity but is limited for quantitative comparisons among cortical sites, subjects, and populations. We have developed a convolutional neural network (CNN) analysis method that learns feature vectors for accurate identification of group differences in fNIRS responses. In this study, subject gender was classified using CNN analysis of fNIRS data. fNIRS data were acquired from male and female subjects during a visual number memory task performed in a white noise environment because previous studies had revealed that the pattern of cortical blood flow during the task differed between males and females. A learned classifier accurately distinguished males from females based on distinct fNIRS signals from regions of interest (ROI) including the inferior frontal gyrus and premotor areas that were identified by the learning algorithm. These cortical regions are associated with memory storage, attention, and task motor response. The accuracy of the classifier suggests stable gender-based differences in cerebral blood flow during this task. The proposed CNN analysis method can objectively identify ROIs using fNIRS time series data for machine learning to distinguish features between groups.

## 1. Introduction

Functional near-infrared spectroscopy (fNIRS) estimates regional cortical activity by measuring local changes in hemoglobin concentration. This neuroimaging modality has numerous advantages including the capacity to measure cortical hemodynamics associated with activity in real time with higher temporal resolution than functional magnetic resonance imaging (fMRI) and positron emission tomography (PET). fMRI measures the hemodynamic response associated with neuronal activity based on nuclear magnetic resonance. PET also detects the brain activity by measuring cerebral hemodynamics and oxygen metabolism. While they have higher spatial resolution than fNIRS, their temporal resolution is poor (e.g., a few seconds for fMRI, minutes for PET, and milliseconds for fNIRS). Furthermore, fNIRS permits a greater range of tasks during acquisition because the measurement diode array is fixed to the subject's scalp. Thus, the problem of movement artifacts is minimal compared to fMRI (but still needs attention). fNIRS presents advantages in its fully noninvasiveness, ease of use, portability, and low cost. These advantages have resulted in broad use of fNIRS for human cognitive studies [1–4].

However, there are also limitations to fNIRS. Activity is measured as a relative change because there is no consistent relationship between cortical activity and local oxy-hemoglobin (oxy-Hb) concentration. In addition, estimation of hemoglobin concentration based on the modified Beer–Lambert law requires knowledge of local optical path length (i.e., the distance from the scalp surface to the cortical surface), which varies with scalp position and among individuals. These limitations make it difficult to compare data from different channels between individuals as well as within the individual. Thus, it is necessary to utilize a summary static approach. Moreover, despite the high temporal resolution, fNIRS data is treated as a feature quantity (e.g., oxy-Hb) for comparison, and all temporal information is lost. Extensive preprocessing of fNIRS data is also required, including correction for motion artifacts and baseline drift (low-frequency fluctuations). Setting parameters for these processes is difficult or arbitrary because the optimal settings differ for each individual subject and task.

However, most current fNIRS studies use dozens of individual channels distributed over a broad region of the scalp, thereby making it possible to perform network analysis between channels or functional connectivity analysis (FCA). FCA is a form of seed-based analysis or independent component analysis. In this case, it is necessary to determine the most appropriate region of interest (ROI) as the seed; however, this decision is also highly subjective.

Resolution of these analytical problems is necessary to fully realize the potential of fNIRS as a noninvasive, safe, and accessible alternative to fMRI for human studies. Among the required developments of seminal importance are the automation of preprocessing and determination of the seed ROI to facilitate group analysis of fNIRS data while retaining the temporal information in the time series acquired from each measurement channel.

Previously, the authors have proposed a gender classification method for fNIRS time series data using deep learning [5], a type of machine learning. In the proposed method, a stacked denoising autoencoder (SDA) [6] and a deep neural network (DNN) are used and trained to classify the gender of a subject from given fNIRS data. One advantage of using deep learning methodology is that it requires minimal preprocessing because optimal settings are learned automatically [7]. Our classifier achieved 81% accuracy for gender classification.

In this study, we focus on another aspect of the deep learning methodology regarding ROI determination. If we can derive the gender classifier for each fNIRS channel, we can determine which channel provides better classification accuracy. These channels are simply the best ROIs for classification/differentiation of the subjects. We apply a convolutional neural network (CNN) [8], which is a type of deep learning method, to construct the gender classifier. One major advantage of CNNs is that feature extraction and classification are integrated into a single structure and optimized automatically. fNIRS time series data of human subjects were input to the CNN, and then the features of the data were learned to classify the gender of the subjects. The proposed CNN-based classifier for automatic determination of the most suitable ROI is described in detail and its performance is verified experimentally. To examine the effectiveness of the proposed method, a simple memory task for visually presented single-digit numbers was performed by adult subjects in a white noise environment. We verify that CNN analysis can identify an ROI (seed region) to distinguish males from females based on differences in the hemodynamic response pattern during the task.

## 2. Materials and Methods

### 2.1. Selection of ROIs for Subject Classification

When we deal with fNIRS data, we often compare them among multiple groups. In this case, a major problem is determining which regions should receive focus. To overcome this issue, we extract ROIs that are prominent to separate human subjects into some groups. As these ROIs maximize the difference between the groups, it will be useful to compare the brain activity among them.

To extract such ROIs, a classifier-based approach is proposed. First, a group classifier is constructed from all subject fNIRS data using supervised learning. The group classifier is constructed for each channel of an fNIRS measurement system, and a group label is supervised during each learning process. After the learning process is completed, the classification accuracy of the classifier for each channel is compared among all channels, and the channel whose classifier has better accuracy is extracted as the critical ROI for group classification. In this study, we use a CNN as the supervised learning algorithm.

### 2.2. Convolutional Neural Network

A CNN is a type of feed-forward artificial neural network that has two hidden layers, convolution and pooling. The weights of these hidden layers within the connected network are learned through repetition.

**Figure 1:**
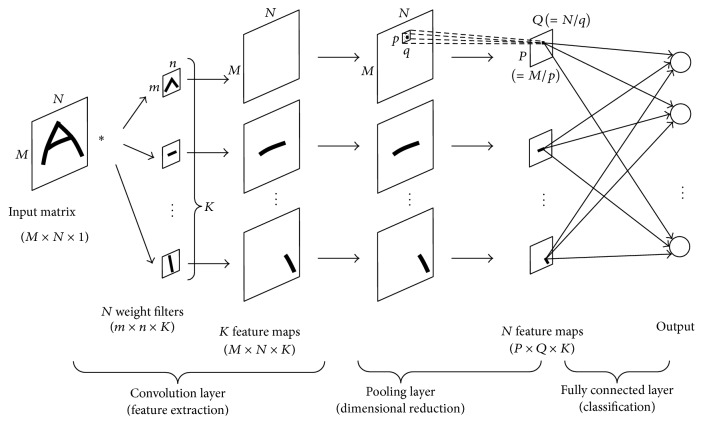
CNN structure.

Figure 1 shows the structure of the CNN used for image recognition. Here *M* × *N* pixel image is input to the convolution layer. In the convolution layer, *K* numbers of feature extraction filters, referred to as “kernels,” are convoluted over the entire image using *m* × *n* window. After convolution between the image and the kernel, the convolution process is formulated as follows:

(1) $$\begin{array}{c} u_{ijk}=\overset{m-1}{\underset{a=0}{\sum }}\;\overset{n-1}{\underset{b=0}{\sum }}x_{\left(i+a\right)\left(j+b\right)}w_{abk}, \end{array}$$

where *x*_*ij*_ is the pixel (*i*, *j*) of the input image matrix, *w*_*ijk*_ is the weight to the pixel (*i*, *j*) of the* k*th kernel, and *u*_*ijk*_ is the convoluted output with the* k*th kernel at the pixel (*i*, *j*).

Then, the convoluted output is processed by the activation function, and this is the final output in the convolution layer:

(2) $$\begin{array}{c} y_{ijk}=f\left(\;u_{ijk}\;\right). \end{array}$$

Finally, *K* feature maps are obtained in the convolution layer. Ordinary artificial neural networks (ANN) require preprocessing of feature extraction before learning; however, a CNN includes the feature extraction process in its architecture. This is the reason that we choose the CNN as the classifier for our problem. As mentioned in Section 1, it is difficult to appropriately preprocess the fNIRS data so as to extract feature value for classification. CNN can automatically do it.

Feature maps obtained through the convolution layer are then processed by the pooling layer. In the pooling layer, extra feature information for learning is discarded. Although several types of pooling are proposed, we use a max pooling method. In this method, *p* × *q* pixels (referred to as pooling size) are extracted from each feature map, and the maximum value is adopted as a single representative output value as follows:

(3) $$\begin{array}{c} y_{cdk}^{\prime }=\underset{\left(a,b\right)\in P_{\mathrm{ijk}}}{\max}y_{ijk}, \end{array}$$

where *P*_*ijk*_ is the pooling block with pixel (*i*, *j*) at the left top of the block in the *k*th feature map. Then, (*M*/*p*) × (*N*/*q*) pixel image is obtained because each *p* × *q* pixel outputs a single output value. In other words, *y*_*cdk*_′ is the output of each pixel (*c*, *d*) in the (*M*/*p*) × (*N*/*q*) image. The features of the image are preserved even in the reduced image size. Several series of convolution and pooling layers can be connected repetitively.

The output layer comprises a fully connected neural network, similar to ordinary ANNs. In the output layer, the number of neurons is equivalent to the number of classes in the data classification. Each neuron in the output layer is connected to all neurons in the previous layer (the final pooling layer), and its output is calculated using (4) and the softmax function of (5):

(4) uj=∑i=1M/p×N/q×Kwijyi′+bj,

(5) pj=euj∑k=1Ceuk,

where *u*_*j*_ is the input to the neuron *j* in the output layer, *y*_*i*_′ is an output of the pooling layer, *w*_*ij*_ is the weight from neuron *i* in the pooling layer to neuron *j* in the output layer, *b*_*j*_ is the bias,* C* is the number of classes in the data classification, and *p*_*j*_ is the probability that the input data is classified to class* j*.

Backpropagation is used to learn the weights. This process is the same as ordinary ANNs. The backpropagation optimizes the weights of each layer to minimize the error between the output of the fully connected layer and the supervised output. By repeating the forward and backward propagation, the CNN is trained to classify the input data.

### 2.3. Gender Classification by Cerebral Blood Flow Changes Using Convolutional Neural Network Analysis

Cortical activity was estimated by multiple fNIRS channels positioned over the left hemisphere. The objective was to classify each human subject as male or female based on the fNIRS response during the task (described in the following section). Therefore, we construct a two-class classifier using the CNN. The CNN can extract the features of small local parts of the input because the plural local filters (kernels) are convolved with the input. The extracted local features are mixed and integrated into a global feature in the fully connected output layer. The SDA and DNN, which have been used in a previous study [5], do not extract local features because they employ a fully connected architecture.

**Figure 2:**
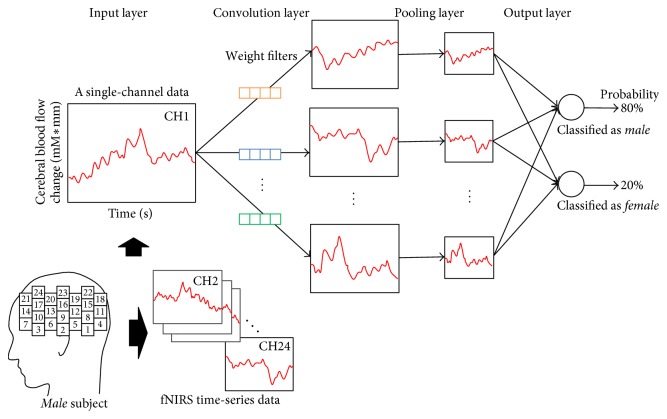
CNN for gender classification by fNIRS data.

In this study, the input data is time series cerebral blood flow change (oxy-Hb versus time) measured at each fNIRS channel, and the CNN is trained to classify the input fNIRS data as either “male” or “female.” The output values of the two neurons in the final layer of the CNN are the probabilities that the input data belong to male or female. In backpropagation, “male” data are supervised as 0, and 1 is used for “female” data. The proposed CNN for gender classification is illustrated in Figure 2. The fNIRS time series data of each channel is 1 × *N* and is input to the convolution layer. The weight filters are initialized randomly and optimized in backpropagation. A single set of convolution and pooling layers is utilized.

After CNN training, we obtain the gender classifier for each fNIRS channel. The average value of the leave-one-out cross-validation for each channel was compared, and the channel that yielded the highest average value was defined as the critical ROI (seed) for generating a label set for the task.* As the convolution is performed in the sliding window manner*,* the feature extraction process of CNN* retains the temporal information of the time series data obtained by fNIRS, which is novel among analysis methods for group trends in fNIRS studies.

### 2.4. Experiments

To confirm the effectiveness of the techniques described in the previous section, the ROI of this task was identified using the CNN by classifying the differences in regional cortical blood flow of male and female subjects during a number memory task.

#### 2.4.1. Experimental Outline

In the number memory experiment, subjects were required to memorize eight single-digit numbers presented on an LCD monitor while white noise was presented (sound pressure level 65.0 ± 0.5 dB) through speakers positioned at the left and right of the display. It has been reported that the cerebral blood flow pattern during this specific task differs between males and females [4].

#### 2.4.2. Task Design

**Figure 3:**
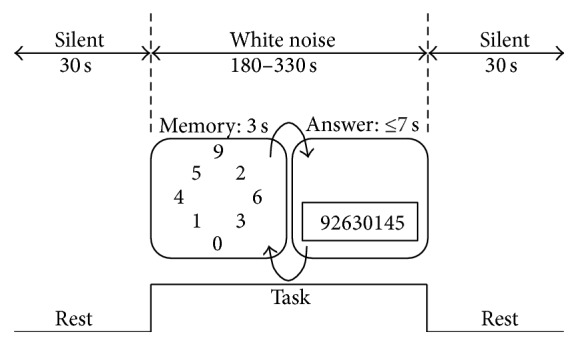
Flow of measurement of the number memory task.

The phases of the task are illustrated in Figure 3. In the rest phase (1), the subjects simply move their fingers while watching a blank screen for 30 s. In the memory phase (2), the subjects memorize eight numbers in 3 s. The numbers were displayed randomly and arranged in a circle. In the retention phase (3), the subjects were required to retain the numbers in memory for 1 s. In the input/retrieval phase (4), the subjects entered the remembered numbers in order (counterclockwise) within 7 s.

The subjects repeated phases (2)–(4) 30 times (phase 5), followed by a final rest phase (6) identical to phase (1). For fNIRS acquisition, the low-pass filter was set to 1.0 Hz, and the period of the moving average was set to 10 s.

#### 2.4.3. Experimental Environment

**Figure 4:**
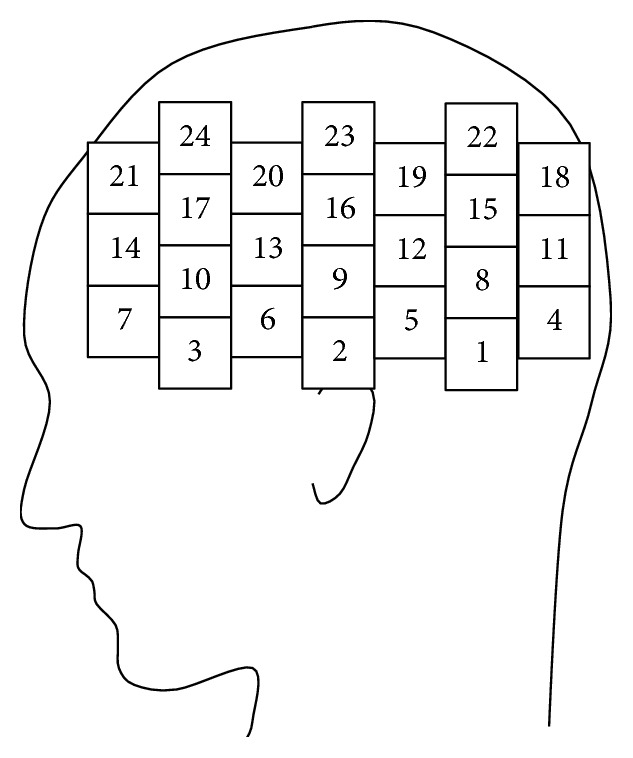
Placement of fNIRS probes (channels) over the left hemisphere.

Cerebral blood flow changes were measured by an fNIRS device (ETG-7100, Hitachi Medical Corporation) at a sampling frequency of 10 Hz. The subjects were 11 adult males (average age: 22.5 ± 0.5 years; all but one were right-handed) and 11 adult females (average age: 22.5 ± 0.5 years). In the measurement environment, the room temperature was 22.4–25.1°C, and the humidity was 40–61%. All trials were conducted between 11 a.m. and 5 p.m. The fNIRS probes were placed according to the International 10–20 system. The scalp sites of the channels (CH) are shown in Figure 4.

#### 2.4.4. Preprocessing of fNIRS Data

**Figure 5:**
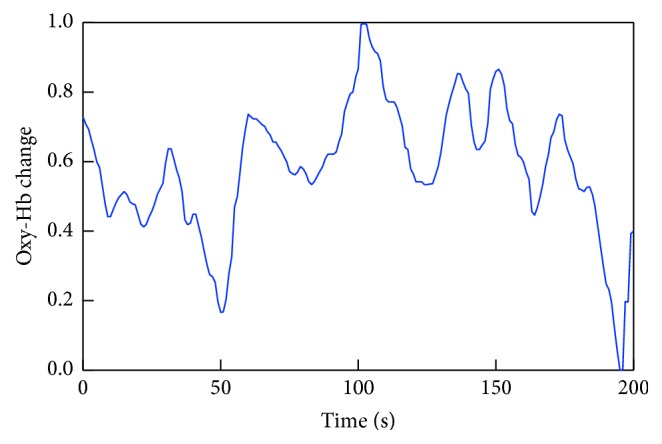
Example of the extracted feature amplitude.

The cerebral blood flow changes were measured by the feature quantity derived from the oxy-Hb concentration changes for each channel over the temporal region extracted for all 22 subjects. As the “feature value,” the average value over 1 s from initiation of the task was used. The signals of each channel were normalized to the min–max and labeled “gender.” An example of the extracted feature amplitude is shown in Figure 5.

#### 2.4.5. Configuration of CNN

**Figure 6:**
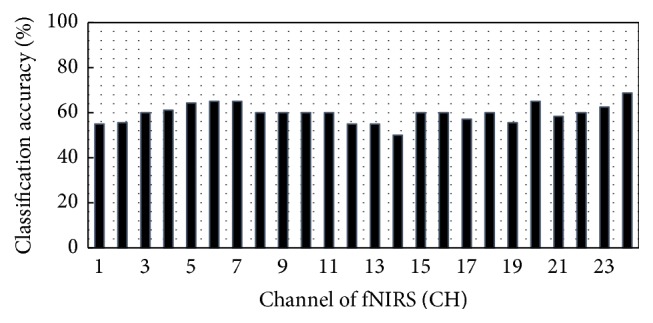
Gender-based differentiation accuracy of each fNIRS channel.

We distinguished males from females using the regional cortical blood flow change feature. Each neuron in the input layer receives the extracted feature value from one channel. The output layer performs learning of the weight so as to approach the labeled value “gender.” The number of samples was 22 (subjects), and the total number of epochs for learning was 5000. We calculated the identification rate for each of the 24 channels (Figure 6). We then identified the channels with the highest discriminating value for males and females (the most reliable difference in the feature quantity). To verify accuracy, leave-one-out cross-validation was performed for all 22 subjects. The parameters of the CNN are shown in Table 1.

## 3. Results and Discussion

Figure 6 shows the gender-based differentiation accuracy for each channel. Each bar is the average value of the leave-one-out cross-validation.

**Figure 7:**
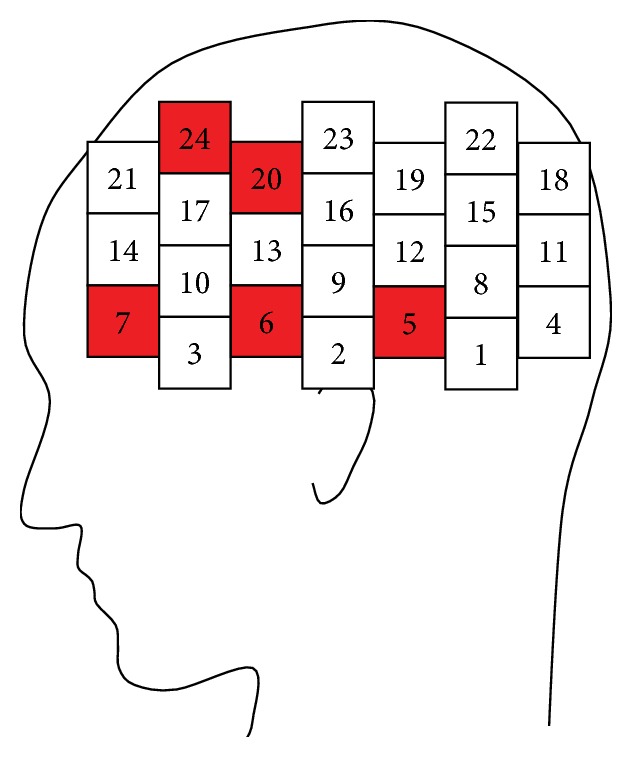
Locations of channels with the highest discrimination accuracy.

The channels with the highest identification rates were 5, 6, 7, 20, and 24 (Figure 7; red). In other words, the cortical regions showing the largest difference in the feature between males and females for this specific task were measured by channels 5, 6, 7, 20, and 24. Channels 6 and 7 were over the inferior frontal gyrus, which is associated with memory and attention. Channels 20 and 24 were located over the premotor area and were presumably activated by the retrieval task (inputting the remembered numbers).

Finally, channel 5 is over the primary auditory cortex and is likely activated by the white noise. Therefore, under white noise sound environment, we suggest that the greatest gender-related differences in cortical activity are related to attention, memory, and task motor response.

**Figure 8:**
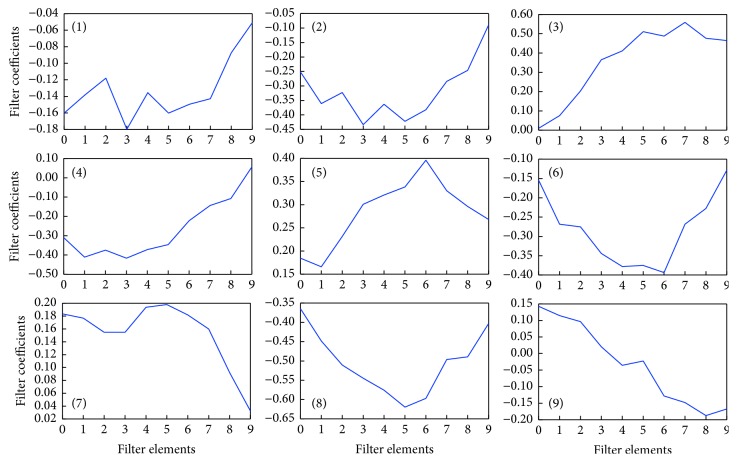
Learned filter properties. Since the filter kernel is 1 × 10, ten temporal elements exist and the output of each element is plotted in the vertical axis.

**Figure 9:**
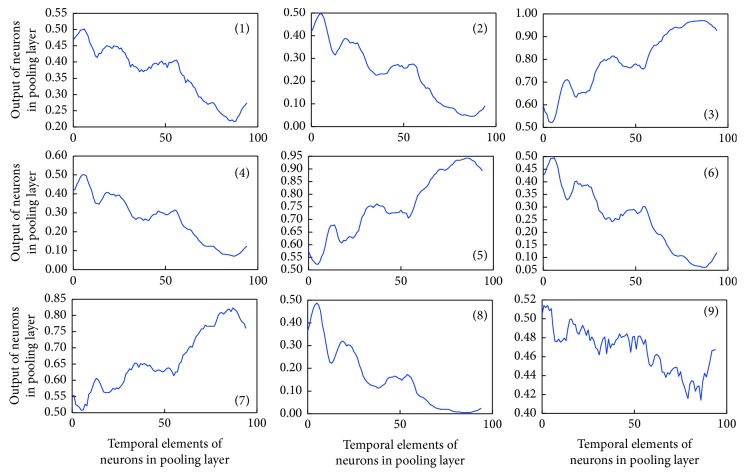
Output of the CNN pooling layer for males. Since the pool shape is 1 × 2, 200-s input data is decomposed into 100 temporal elements (100-s data) after the pooling.

**Figure 10:**
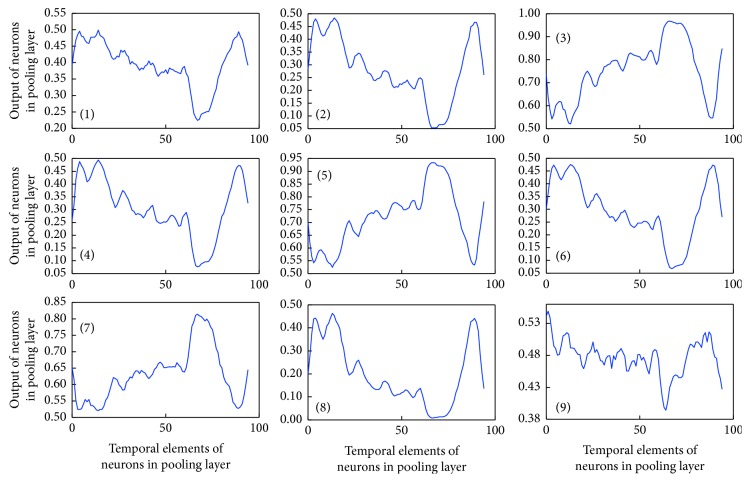
Output of the CNN pooling layer for females.

Next, we assessed the structure of the time series epoch with the highest identification rate by the network.  Figure 8 shows the filters derived after learning. In Figure 8, (1)–(9) represent the filters. Each converts the feature quantity of the input data and passes the converted data to the pooling layer. Figures 9 and 10 show examples of the output results of the pooling layer for males and females, respectively.

**Figure 11:**
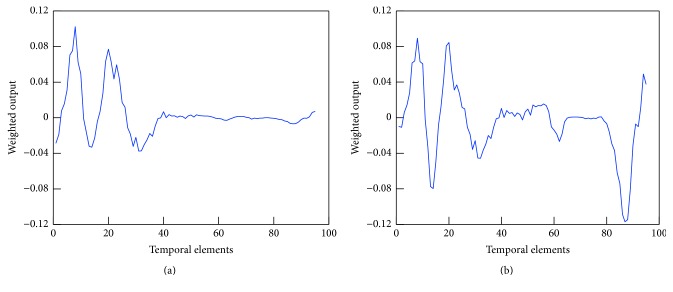
Output of the pooling layer multiplied by filter weight of fully connected layer. There is a clear difference between male and female subjects ((a) men; (b) women).

Filter 9 in Figures 9 and 10 shows the greatest tendency to differ between genders. Finally, Figure 11 shows the results obtained by multiplying the weight of the fully connected layer to the output of the pooling layer for filter 9.

**Figure 12:**
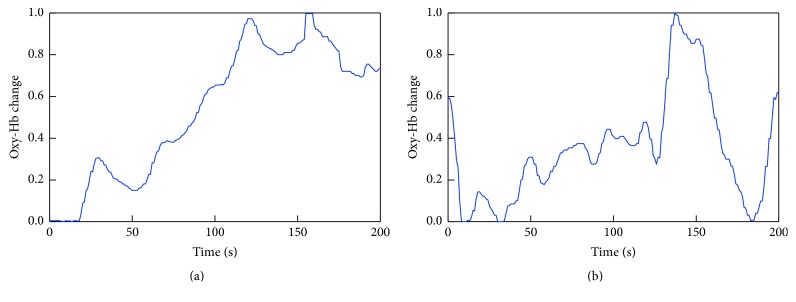
Input data ((a) male; (b) female).

Figure 11 shows a clear difference in the output between male and female subjects. Although no significant difference was observed between the genders in the first half of the task period, the feature quantity for men was close to 0 in the latter half, while that for females decreased rapidly.  Figure 12 shows the input data for males and females. From Figures 11 and 12, it is clear that the CNN was modified to allow classification of males and females based on the unique cerebral blood flow patterns during the task.

## 4. Conclusion

In this study, we have performed a preliminary evaluation of a CNN-based method for automatic determination of the ROI for fNIRS group analysis. The proposed method retains the temporal information of the fNIRS data in contrast to conventional summary static approaches for group-level analysis.

We propose that this method for determining the critical ROI for a given task by learning the identity of the subject labels could be employed for revealing unexpected differences between groups in fNIRS data. The effectiveness of the proposed method has been confirmed using a visual number memory task under a white noise sound environment, which is a task known to induce distinct hemodynamic changes in males and females. An ROI was established by learning the feature value. In this task, the inferior frontal gyrus, premotor area, and primary auditory cortex were extracted as the ROI, which are sites related to memory storage, attention, answer output, and detection of white noise. Gender differences in hemodynamic response in this ROI were identified accurately during the second half of the task. Further work is required to reveal why this task evokes distinct hemodynamic responses in males and females.

## Figures and Tables

**Table 1:** Configuration of CNN.

Type	Parameter
Learning rate	0.05
Momentum	0.998
Kernel shape	1 × 10
Kernel stride	1
Pool shape	1 × 2
Pool stride	1
